# Lithia Waters

**Published:** 1899-02

**Authors:** 


### LITHIA WATERS.

Satisfactory progress in rational therapeutics can only be attained when there is accurate knowledge as to the nature of the materials used, and substantial constancy in the composition of the preparation. One of the most serious objections to secret remedies is the impossibility of being assured as to their nature. The preference given to active principles over the old-fashioned crude drugs is also based on this principle. When, therefore, physicians use natural products as therapeutic agents, there will be no certainty as to cause or constancy of results unless analysis shows the general character of such products, and proper supervision prevents the sale of those which do not contain the active ingredients.

The use of so-called lithia waters is one of the petty disgraces of practical therapeutics. It is not unlikely that the value of medicinal ingredients in water has been much exaggerated. Indeed, a part of the popularity of some springs is doubtless a relic of ancient superstition, under the influence of which a spring was supposed to be the abode of a divinity. The lithia-water theory has, however, been developed in recent years; it claims descent from science, not from superstition. We do not care to deal here with the question whether lithium carbonate will prevent the precipitation of uric acid more effectually than other carbonates; this may be assumed for the purpose of argument. When, however, lithia waters are given for such purpose, the chances are that the patient will get at best but little of the remedy intended, for most of the natural waters sold under that title have but little lithium in them. In one which was extensively advertised as very rich, tests failed to show any appreciable amount, and other kinds in the market contain but minute amounts of lithium compounds. Uric acid requires about half its weight of lithium carbonate to form a complete salt; taking into consideration the average amount of uric acid excreted in twenty-four hours, it is obvious that no distinct neutralizing action is to be expected from the administration of waters which contain less than a grain of the lithium salt to the gallon.

Many physicians undoubtedly prescribe the water without giving the chemical features any thought, or without taking the trouble to find out if the claims made on the label or in the advertisement are true. That good results occasionally follow such treatment is doubtless due to the fact that the water is pure and used freely. If lithium salts are indicated they should be given as such, and the superstitious veneration for a natural water should not be allowed a place in modern therapeutics. Natural waters, rich in lithium, are known, but several contain so much other mineral matter as to make them unsuited for free use.—*Editorial, Philadelphia Polyclinic.*

